# Neural correlates of motor imagery and execution in real-world dynamic behavior: evidence for similarities and differences

**DOI:** 10.3389/fnhum.2024.1412307

**Published:** 2024-06-21

**Authors:** Magda Mustile, Dimitrios Kourtis, Martin G. Edwards, David I. Donaldson, Magdalena Ietswaart

**Affiliations:** ^1^Department of Psychology, Faculty of Natural Sciences, University of Stirling, Stirling, United Kingdom; ^2^The Psychological Sciences Research Institute, University of Louvain, Louvain-la-Neuve, Belgium; ^3^School of Psychology and Neuroscience, University of St Andrews, St. Andrews, United Kingdom

**Keywords:** motor imagery, simulation, brain oscillations, cognitive processes, EEG, functional equivalence

## Abstract

A large body of evidence shows that motor imagery and action execution behaviors result from overlapping neural substrates, even in the absence of overt movement during motor imagery. To date it is unclear how neural activations in motor imagery and execution compare for naturalistic whole-body movements, such as walking. Neuroimaging studies have not directly compared imagery and execution during dynamic walking movements. Here we recorded brain activation with mobile EEG during walking compared to during imagery of walking, with mental counting as a control condition. We asked 24 healthy participants to either walk six steps on a path, imagine taking six steps, or mentally count from one to six. We found beta and alpha power modulation during motor imagery resembling action execution patterns; a correspondence not found performing the control task of mental counting. Neural overlap occurred early in the execution and imagery walking actions, suggesting activation of shared action representations. Remarkably, a distinctive walking-related beta rebound occurred both during action execution and imagery at the end of the action suggesting that, like actual walking, motor imagery involves resetting or inhibition of motor processes. However, we also found that motor imagery elicits a distinct pattern of more distributed beta activity, especially at the beginning of the task. These results indicate that motor imagery and execution of naturalistic walking involve shared motor-cognitive activations, but that motor imagery requires additional cortical resources.

## 1 Introduction

The term motor imagery has been used in literature to indicate the visual (i.e., imagine to “see”) and kinaesthetic (i.e., imagine to “feel”) imagination of a movement without execution (Jeannerod, 1994, 2001; Decety, 1996; Mulder, 2007). Since the first seminal studies in the 1930s (Sackett, 1934, 1935), a large body of evidence shows that motor imagery and actual action execution share similar cognitive and neural processes. For example, the time taken to mentally imagine versus execute a movement has been shown to be similar (Decety and Michel, 1989; Sirigu et al., 1995) suggesting that imagery is based on overlapping action execution cognitive processes. Similarly, execution and motor imagery both adhere to Fitts’s law, whereby the time to execute or imagine a movement is moderated by accuracy demands (Decety and Jeannerod, 1995; Macuga et al., 2012; Macuga and Frey, 2012). According to the “functional equivalence hypothesis” (following motor simulation theory, *cf.* Jeannerod, 1994, 2001), similarities between actual execution and motor imagery emerge from shared motor-cognitive (neural) processes, allowing for the imagined rehearsal of movement using cognitive motor planning processes (Jeannerod, 2001). The functional equivalence hypothesis has received strong support, primarily from brain imaging studies which have demonstrated that motor imagery and actual motor execution involve similar activation of brain areas (Porro et al., 1996; Grezes and Decety, 2001; Sharma and Baron, 2013). These brain activations include a distributed premotor-parietal network, involving several subcortical structures, such as the putamen and cerebellum (Grezes and Decety, 2001; Hardwick et al., 2018). Further indirect evidence for equivalence comes from sport (Guillot and Collet, 2008; Williams et al., 2012) and clinical literature, which have shown that the mental practice of movement through motor imagery (i.e., the repetitive exposure to motor imagery of movements) can be effective for learning motor skills (Dijkerman et al., 2010; Barclay et al., 2020). The potential efficacy of mental practice in sport training and in motor rehabilitation is supported by the finding that motor imagery practice can induce plastic changes in motor networks (for a review see Ruffino et al., 2017).

Despite this common notion of the evidence for functional equivalence, technical constraints make measures of motor imagery extremely challenging for researchers, even in experimentally controlled settings. Motor imagery is a covert process. This means that, without comparison to actual execution, it is not always clear what is being measured by recording brain activations during motor imagery. Furthermore, neural measurement during movement execution is also limited, in particular for whole-body movements such as walking (Ladouce et al., 2017). Understanding the motor imagery of walking requires capturing the neural processes during whole-body movements, which cannot be done while lying in a brain scanner (nor while seated in a typical neurophysiology lab). Instead, researchers investigating functional equivalence between motor imagery and execution have constrained actions to simplistic hand movements that are feasible in an fMRI scanner. A further constraint is that imagined movements in experimental fMRI studies are performed lying down, whereas the imagined movements are more normally performed upright, or sitting (Jahn et al., 2004, 2008; Bakker et al., 2007; La Fougere et al., 2010; Malouin et al., 2013; Hamacher et al., 2015). It is possible that incongruence between body posture and imagined action causes an issue for ecological validity, also because the compatibility of the body position has been shown to affect motor imagery performance. For example, the time taken to perform motor imagery is most similar to actual execution when participants are positioned in a congruent compared to incongruent posture for the imagined relative to executed movement (Parsons, 1994; de Lange et al., 2006; Conson et al., 2011; Saimpont et al., 2012). These latter findings are important, as investigating the neural substrate and cognitive processes underlying motor imagery and action execution must be investigated using congruent and equivalent complex motor actions (Barton and Pretty, 2010; Menicucci et al., 2020).

Motor imagery is an established method of upper and lower-limb neuro-rehabilitation used with patients after stroke (Barclay et al., 2020). Considering that deficits of lower limb function are a common disability after stroke, it is surprising that there is just one single study to date that compares the neural activation of the execution and motor imagery of walking (La Fougere et al., 2010). They captured global neural activation during walking using PET imaging, and compared neural activation to motor imagery of walking using fMRI while lying flat. La Fougere et al. (2010) showed PET neural activation during walking execution involved primary motor cortex and somatosensory activation, while the fMRI neural activation measured during motor imagery of walking involved the activation of the supplementary motor area and basal ganglia. There are, however, some fundamental limitations to that study, in particular with regards to the design. Firstly, the measures of PET and fMRI are quite different, and it is not clear why the neural correlates of execution and motor imagery in that study were not measured using the same imaging methods allowing direct comparison. Secondly, activation of execution was captured through isotopic decay measured in the PET scanner after people had been walking. While ingenious as a solution and to be admired in its ambition as an early attempt of neuroimaging of real-world whole-body movements, the neural correlates are difficult to compare to motor imagery assessed through fMRI *while* the participant is performing the imagery. Thirdly, as stated above, differences are expected in brain imaging investigations of motor imagery where participants lay supine imagining the upright movement of walking. In conclusion, while groundbreaking, in many ways, the study of La Fougere et al. (2010) provided limited insight into the neural correlates of whole-body motor imagery functional equivalence.

Recent development of mobile electroencephalogram (EEG) technology solves many of the constraints discussed above, including the ability to record brain activity during naturalistic movement. EEG recordings allow a characterization of highly accurate temporal brain rhythm dynamics that reflect cognitive processing, overcoming many of the limitations of functional MRI scanning. Furthermore, the high temporal accuracy of EEG offers the possibility to disentangle cognitive processes occurring during motor imagery and action execution at different time points. It is crucial to investigate the neural correlates of these processes considering the growing applications of motor imagery in clinical settings. Indeed, motor imagery of movements represents one of the main cognitive tools being used within the brain-computer interface (BCI) approach, as it can be applied in absence of physical involvement and can be employed as a self-regulatory control signal of motor brain areas. The principle of the BCI approach is to detect neural signals to control external devices, such as exoskeletons or wheelchairs (Leeb et al., 2007; Choi and Cichocki, 2008; Lafleur et al., 2013) or prostheses (Pfurtscheller et al., 2003). It is therefore important to understand which are the neural correlates of walking imagery as they can be used as self-regulatory signals for motor learning and recovery in patients with gait impairments (Malouin and Richards, 2010; Kranczioch et al., 2014; Daeglau et al., 2020).

EEG investigations of motor imagery in isolated movements such as those of the foot or the hand have found decreased and increased spectral power, often termed event related desynchronization and synchronization, respectively. These changes in power are most often found in the alpha (8–12 Hz) and beta (13–35 Hz) frequency bands, occurring over sensorimotor (Pfurtscheller et al., 1997; Pfurtscheller and Neuper, 1997; Neuper and Pfurtscheller, 2001) and parietal-occipital (Salenius et al., 1995; Xie et al., 2020; Putzolu et al., 2022) brain areas. Generally, it is accepted that power decreases in the alpha and beta frequency bands are related to the activation of relevant brain areas during a given task, whereas power increases are associated with inhibition (Pfurtscheller, 1999; Pfurtscheller and Da Silva, 1999; Klimesch, 2012). Indeed, both alpha and beta decrease of power over sensorimotor areas are typically visible during movement preparation (Tzagarakis et al., 2010; Rhodes et al., 2018), the execution of movements (Pfurtscheller and Berghold, 1989; Stančák et al., 1997; Cassim et al., 2000; Kaiser et al., 2003; Kilavik et al., 2013) and have been consistently reported in previous EEG investigations of walking (Gwin et al., 2011; Bulea et al., 2015; Bradford et al., 2016). On the other hand, power increases in the alpha band have been associated with the inhibition of task-irrelevant information (Neuper et al., 2006; Klimesch, 2012), whereas beta power increases are associated with the recalibration of the motor system (Engel and Fries, 2010). Although previous EEG investigations have provided evidence of the engagement of sensorimotor brain areas during action execution and motor imagery, research has typically focused on upper limb movements, while participants were sitting, performing small hand or finger movements (e.g., finger-tapping). Studies of lower limb imagery were mostly limited to imagery of minimal movement, such as the dorsiflexion of the foot (Neuper et al., 2010; Pfurtscheller et al., 2006; Solis-Escalante et al., 2008, 2012; Müller-Putz et al., 2010; Hashimoto and Ushiba, 2013), stepping in place (Kline et al., 2021), or they did not contrast cortical activation with the actual execution of movement (Putzolu et al., 2022). Furthermore, existing EEG investigations on motor imagery have tended to focus on identifying reliable signals for brain-computer interface (BCI) control as mentioned above, allowing for connection between the oscillatory activity of the brain to control a computer (Wolpaw et al., 2002). Consequently, EEG recordings in these studies have typically involved a limited number of channels (leaving cognitive questions less explored). No existing studies report a systematic assessment of similarities and differences in brain activity and cognitive mechanisms underlying the actual execution and motor imagery of walking.

Therefore, the aim of the present study was to investigate the similarities and differences of neural activations associated with action execution and motor imagery of walking movements using a mobile EEG approach. In a within-subject study design, we asked participants to walk, or to imagine walking (in a movement compatible standing position), at a natural pace, along a path. To enhance the sensorimotor experience, we asked participants to perform the imagery straight after walking, using a first-person perspective and focussing on the kinaesthetic experience (feeling the sensations as if actually walking), which has been shown to elicit greater motor activation compared to visual motor imagery from a third person perspective (Lorey et al., 2009; Mizuguchi et al., 2017). Furthermore, we used a mental counting task as a non-motor control condition to ensure that during imagery, participants were not just counting steps, but instead actually imagined themselves walking. We assessed similarities and differences between motor imagery and actual execution by comparing the temporal and spatial features of EEG patterns. Specifically, we examined the time course and the spatial distribution of power spectral changes in alpha and beta frequency bands, according to previous literature on motor imagery (Pfurtscheller et al., 1997; Pfurtscheller and Neuper, 1997; Neuper and Pfurtscheller, 2001).

## 2 Methods

### 2.1 Participants

A total of 24 healthy participants (23 female; age range = 18–44 years; mean = 22.16 years, SD = 6.8 years) took part in the experiment. Sample size was determined by research on relevant literature (see Hashimoto and Ushiba, 2013; Kline et al., 2021; Putzolu et al., 2022). The data from three participants were excluded due to the presence of prominent artifacts in EEG recordings. The remaining data of 21 participants (20 female; age range = 18–44 years; mean = 21.43 years, SD = 5.5 years) were included in the reported analyses. Height (169.05 ± 8.02 cm), weight (67.5 ± 15.39 Kg) and walking speed (5.29 ± 0.56 Km/h) were also recorded for each participant. The participants had no history of neurological disorders and were right-handed (self-reported). Before the experiment, all the participants gave written informed consent. Ethical approval was provided by the local research ethics committee.

### 2.2 Materials and procedure

Participants were asked to complete four experimental conditions: (i) Actual walking, (ii) Motor Imagery of walking, (iii) Mental Counting, and (iv) Observation of walking. In this study, we focus on the comparisons between Motor Imagery, Actual Execution and Mental Counting; the analyses related to action observation are reported in a separate article (Mustile et al., 2022). Participants completed a total of 120 trials (40 trials for each condition) divided into 6 experimental blocks of 20 trials each. During the mental counting (MC) condition, participants were standing and instructed to listen to 6 consecutive tones (107 dB, interval of 600 ms), and then to mentally count up to six using the same time frequency. They were asked to say out loud “six” when they finished the mental count. During the execution of walking (EXE) condition, participants were instructed to take 6 steps on a 6 m long carpet. They walked up and down the carpet without stopping. A laser beam at each end allowed trials to be separated so that just taking the six steps would amount to one trial in the EXE condition (turns were not included). During the motor imagery of walking (MI) condition, participants were instructed to take 6 steps, stop at the beginning of the 6 m carpet and then imagine feeling the sensation as if they were walking down the carpet by mentally performing 6 steps and “arrive” at the end of the path using motor imagery. They were asked to say out loud “stop” when they finished the sixth step of the mental walking task. One motor imagery trial would be the period between coming to a standstill to start the motor imagery and the time the participant said “stop.” To ensure the success of the task, participants were first trained for several minutes. During training, participants were first introduced to the kinaesthetic imagery perspective: an explanation of kinaesthetic imagery as the imagination of “feeling” the sensation of walking without moving and were given the opportunity to practice motor imagery through some examples in order to ensure that they understood the task. Secondly, they were told to concentrate their attention on the movement of their legs, and the “feeling” of pushing their foot on the ground, focusing on the sensation coming from their different muscles of the lower limbs. They were asked to try for several minutes before starting the block of MI, until they were certain that they would be able to perform the task. Depending on the condition, a trial was defined as the time period from when the participants started to mentally count until they said “six” (MC condition), from when they started to walk (6 steps) until the end of the path (EXE condition) and from when they started the imagery of walking until they said “stop” (MI condition). The order of conditions and experimental blocks were randomized across participants.

### 2.3 EEG recording and processing

EEG data were recorded from 32 Ag/AgCl electrodes connected to a portable amplifier (ANT-neuro, Enschede, Netherlands). Electrodes were positioned according to the International 10–20 system: FP1, FPz, FP2, F7, F3, Fz, F4, F8, FC5, FC1, FC2, FC6, M1, T7, C3, Cz, C4, T8, M2, CP5, CP1, CP2, CP6, P7, P3, Pz, P4, P8, POz, O1, Oz, O2. AFz electrode was used as ground and CPz electrode was used as reference. The electrode impedances were reduced below 5 kΩ before the recording. During recording, EEG data were sampled at 500 Hz and bandpass filtered at 0.01–250 Hz. EEG data analyses were performed using custom scripts written in MATLAB 2019a (The MathWorks) incorporating EEGLAB toolbox (Delorme and Makeig, 2004). Data from the mastoid channels (M1 and M2) were removed from the analysis, and all remaining EEG data were filtered using a 0.1–40 Hz bandpass filter. EEG channels with prominent artifacts were automatically selected (kurtosis >5 SDs) and interpolated, and all channels were then re-referenced to the average. Data were downsampled to 250 Hz and an extended infomax Independent Component Analysis (ICA) (Makeig et al., 1995) was performed to identify and remove non-brain signals. Brain-related-ICs were identified using the IClabel plugin (Pion-Tonachini et al., 2019). Components exceeding a 90% probability of being eye, muscle, heart, line noise, and channel noise were rejected. Only brain ICs with dipoles located inside the head and a residual variance lower than 15% were kept. An average of (mean ± SD) 6.65 ± 0.81 ICs across conditions was retained for the analysis.

### 2.4 EEG analysis

**Figure 1:**
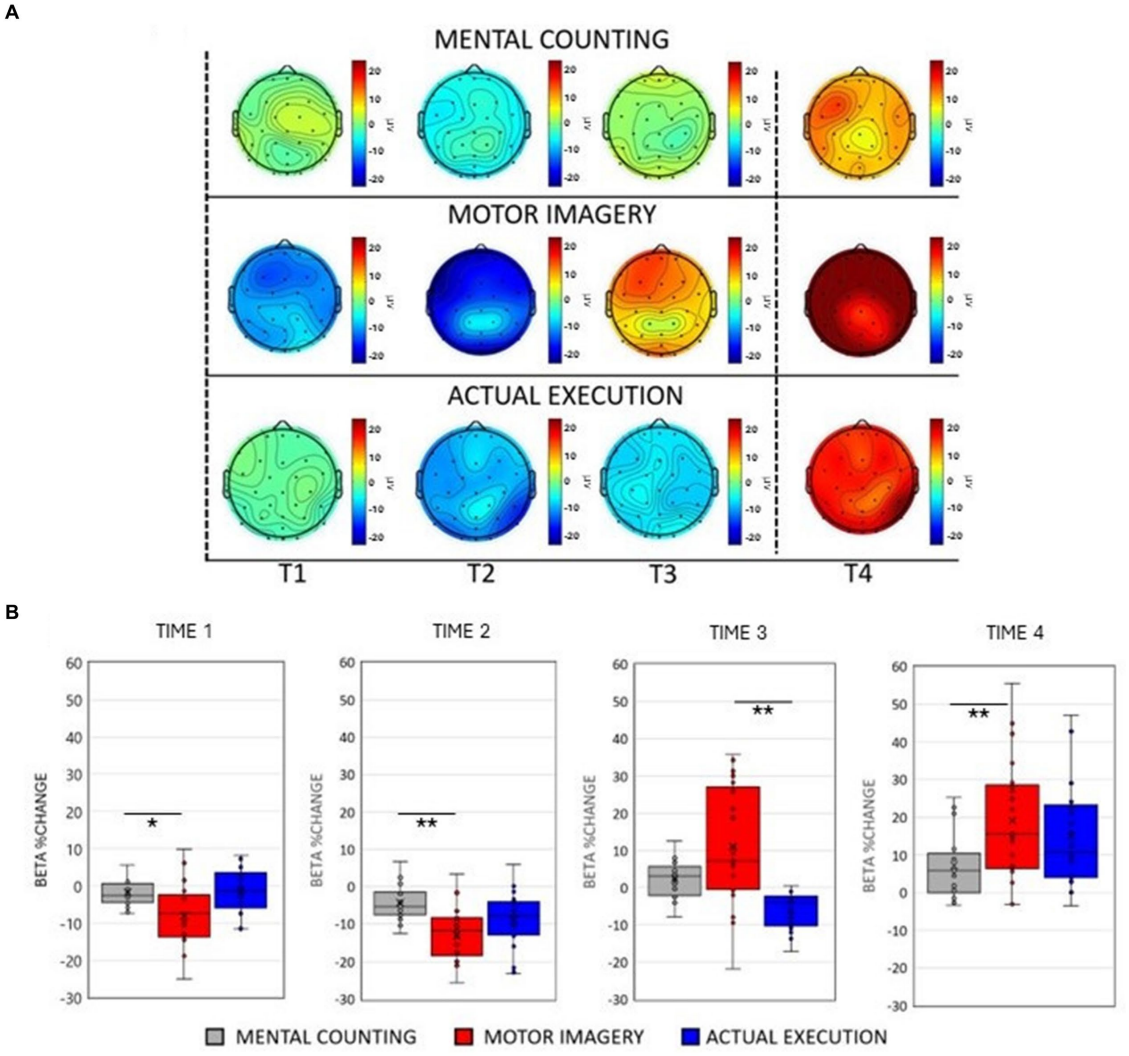
**(A)** Scalp maps topography of beta (13–25 Hz) spectral power across conditions and time. Dashed black lines represent the start and the end of the trial. **(B)** Averaged beta percentage change of power relative to baseline pooling the ROIs (central, parietal and occipital) for each condition in each time window. Significant comparisons are flagged with an asterisk.

**Figure 2:**
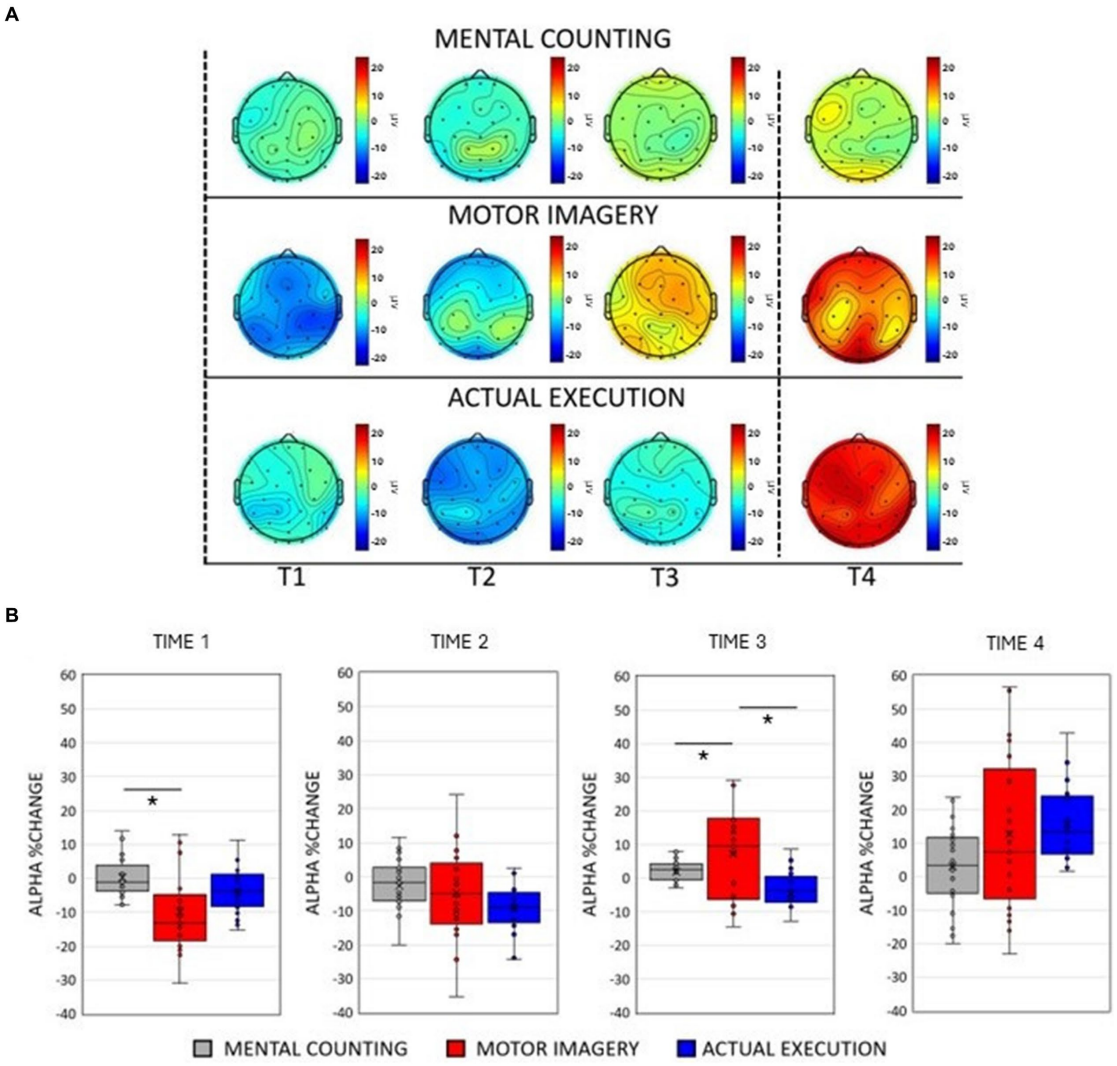
**(A)** Scalp maps topography of alpha (8–12 Hz) spectral power across conditions and time. Dashed black lines represent the start and the end of the trial. **(B)** Averaged alpha percentage change of power relative to baseline pooling the ROIs (central, parietal and occipital) for each condition in each time window. Significant comparisons are flagged with an asterisk.

To investigate the cortical dynamics during the overall length of trials across conditions, EEG data were segmented in epochs of 8,500 ms. An epoch lasted from −7,000 ms before the end of a trial to 1,500 ms after for each condition. Single channel spectrograms were time warped to the median latency of the start of the trial across participants for each condition (−3,826 ms for MC, −3,934 ms for MI, −3,522 for RW, respectively). Event related spectral perturbation (ERSPs) was computed as the mean difference between single trial log spectrograms for each channel and each participant across conditions and the mean baseline (−4,000 ms before to 1,500 ms after time 0). Single channel time frequency spectrograms were visually inspected to identify relevant changes in the spectral power in the *a priori* defined frequency bands of interest: alpha (8–12 Hz) and beta (13–35 Hz). Topographical scalp maps in the frequency bands of alpha and beta (Figures 1, 2A) were further visually inspected to identify relevant regions of interest (ROI). We identified central (FC1, FC2, C3, C4, CZ), parietal (CP1, CP2, P3, P4, PZ channels) and occipital (O1, O2, OZ, POZ) ROIs.

### 2.5 Statistical analysis

Topographic scalp maps (Figures 1, 2A) showed prominent changes in the spectral power in the alpha and beta frequency bands across conditions. To compare power spectral changes across conditions, we first divided the time warped epochs (from the start of the trial until the end) for each condition into three time windows (corresponding to the 33.3% of each epoch). To further examine power spectral changes occurring at the end of the trial, an additional time window of the same proportion was added for each condition after time 0, resulting in 4 time windows. Two separate repeated measures 3 × 4 × 3 ANOVAs with factors of Condition [Mental counting (MC); Execution (EXE); Motor Imagery (MI)], Time (Time 1, Time 2, Time 3, Time 4) and ROIs (central, parietal, occipital) were performed for alpha and beta frequency bands. The Greenhouse—Geisser correction was applied whenever the sphericity assumption was violated and post-hoc paired sample t-tests (adjusted using Bonferroni correction) were performed to further investigate significant main effects and interactions.

## 3 Results

### 3.1 Beta oscillations

The ANOVA revealed a main effect of Condition [*F*(2, 20) = 33.559, *p* <0.001, η*_p_*^2^ = 0.591] showing an overall smaller amplitude in the beta frequency band during execution compared to mental counting [*t*(20) = 3.987, *p* < 0.001] and motor imagery [*t*(20) = 8.192, *p* < 0.001] and an overall larger amplitude of beta in motor imagery compared to mental counting [*t*(20) = 4.204, *p* < 0.001]. A main effect of Time [*F*(1, 20) = 39.841, *p* < 0.001, η*_p_*^2^ = 0.344] revealed that there was a decrease of beta power relative to the baseline in Time 1 and 2, whereas there was an increase of beta power in Time 3 and 4.

As shown in Figures 1A,B, there was an initial beta power decrease during motor imagery and execution, which was evidently different to the modulation of beta power during mental counting. A significant 2-way interaction between Condition and Time [*F*(2, 20) = 8.877, *p* < 0.001, η*_p_*^2^ = 0.287, see Figure 1B], further investigated with post-hoc paired t-tests, confirmed that the decrease in beta power was significantly stronger during the motor imagery compared to mental counting in Time 1 [*t*(20) = 2.634, *p* = 0.048] and Time 2 [*t*(20) = 4.679, *p* < 0.001] but no difference between motor imagery and execution in Time 1 and 2 (*p* > 0.05). In addition, the increase in beta power in Time 4 was significantly stronger during motor imagery compared to mental counting [*t*(20) = 3.236, *p* = 0.002]. With regard to the comparison between motor imagery and execution, there was only a significant difference in Time 3 [*t*(20) = 4.262, *p* < 0.001], due to the earlier appearance of the increase in power in motor imagery.

Although not related to condition, a significant 2-way interaction between ROI and Time [*F*(2, 20) = 11.019, *p* < 0.001, η*_p_*^2^ = 0.015] indicated a different modulation across brain areas during the tasks. Post-hoc paired sample t-tests showed that the beta power decrease was stronger in the central ROI compared to the occipital ROI in Time 2 [*t*(20) = 3.828, *p* = 0.012] and Time 3 [*t*(20) = 3.846, *p* = 0.010]. In addition, there was a stronger decrease of beta power in the parietal ROI compared to the occipital ROI in Time 2 [*t*(20) = 5.481, *p* < 0.001] and a stronger decrease in Time 3 [*t*(20) = 5.749, *p* < 0.001].

There was a significant interaction between Condition and ROI [*F*(2, 20) = 3.749, *p* = 0.031, η*_p_*^2^ = 0.153]. Post-hoc paired t-tests revealed larger amplitudes of beta power in motor imagery compared to execution in the central [*t*(20) = 6.631, *p* < 0.001], parietal [*t*(20) = 5.857, *p* = 0.028] and occipital [*t*(20) = 8.452, *p* < 0.001] ROIs. Similarly, beta power amplitude was larger in motor imagery compared to mental counting over central [*t*(20) = 3.977, *p* = 0.003], parietal [*t*(20) = 3.250, *p* = 0.028] and occipital areas [*t*(20) = 3.520, *p* = 0.013].

**Figure 3:**
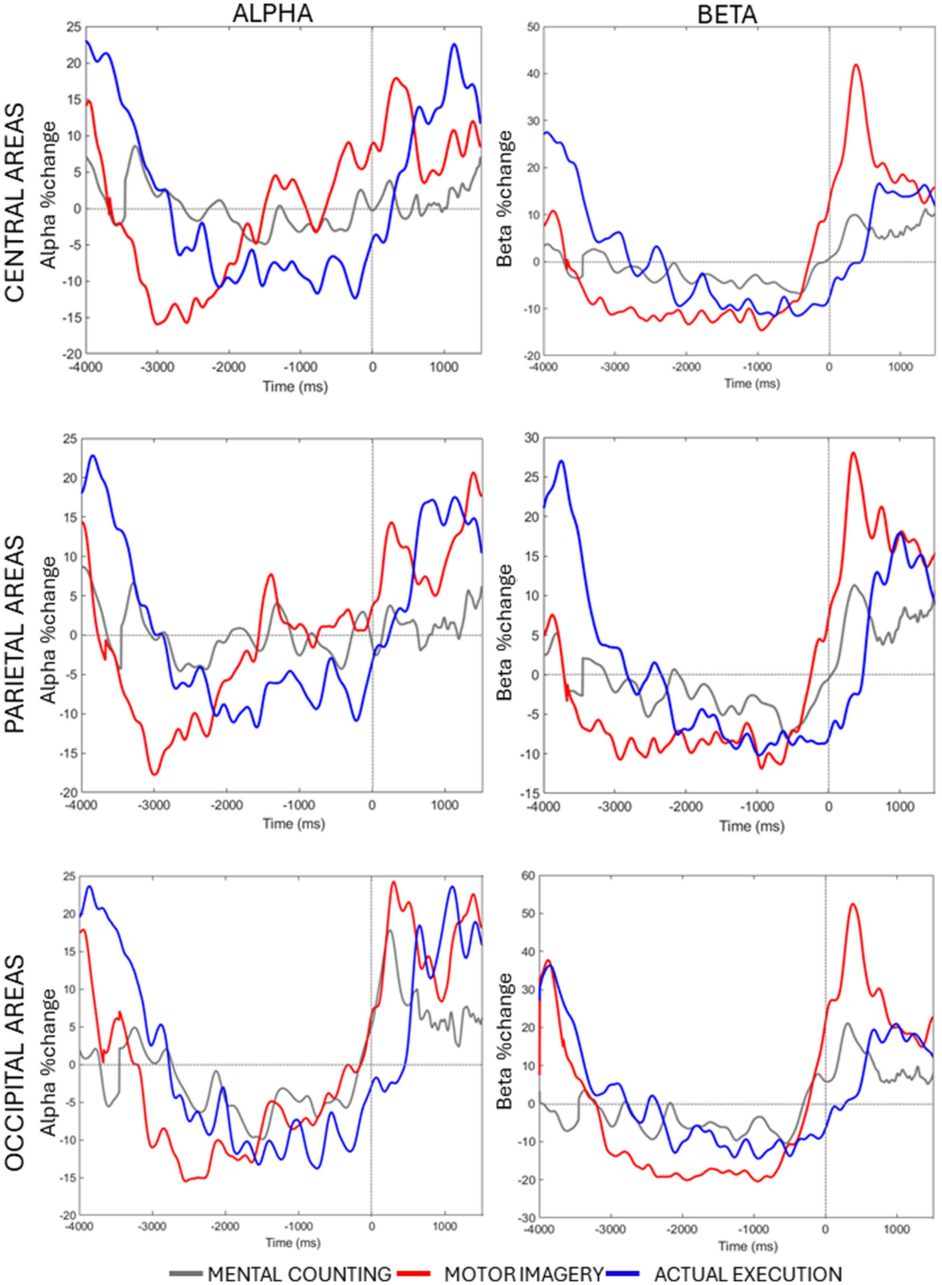
Averaged alpha and beta percentage change of power from baseline across conditions and time in each ROI.

Lastly, a significant 3-way interaction [*F*(2, 20) = 2.990, *p* = 0.033, η*_p_*^2^ = 0.130] was investigated for each ROI separately. Figure 3 (right panels) shows beta power modulation across conditions and time in each ROI. In the central ROI, there was a stronger decrease of beta power in motor imagery compared to mental counting in Time 1 [*t*(20) = 5.965, *p* < 0.001] and Time 2 [*t*(20) = 4.858, *p* < 0.001]. In Time 3 and 4, the beta power increased in relation to the baseline in motor imagery compared both to mental counting [*t*(20) = 3.774, *p* = 0.016] and execution [*t*(20) = 5.897, *p* < 0.001]. In the parietal ROI, the beta power increased in relation to the baseline in Time 3 in motor imagery, whereas there was still a beta decrease in execution; this difference was significant [*t*(20) = 5.209, *p* < 0.001]. In the occipital ROI, there was a stronger decrease of beta power in Time 2 in motor imagery compared to mental counting [*t*(20) = 3.473, *p* = 0.026], whereas there was a stronger increase of beta power in Time 4 in motor imagery compared to mental counting [*t*(20) = 3.938, *p* = 0.009]. In addition, in Time 3, the beta power increased relative to the baseline for motor imagery, but decreased for execution, showing a statistically significant difference [*t*(20) = 5.493, *p* < 0.001].

### 3.2 Alpha oscillations

The ANOVA revealed a main effect of Condition [*F*(2, 20) = 9.917, *p* < 0.001, η*_p_*^2^ = 0.002]. *Post-hoc* paired sample *t*-tests showed an overall smaller amplitude during execution compared to mental counting [*t*(20) = 2.595, *p* = 0.026] and motor imagery [*t*(20) = 4.432, *p* < 0.001], but no differences between MI and MC (*p* > 0.05). A main effect of Time [*F*(2, 20) = 15.896, *p* < 0.001, η*_p_*^2^ = 0.188] revealed that there was a decrease of alpha power in Time 1 and 2, whereas there was an increase of alpha power in Time 3 and 4.

As illustrated in Figures 2A,B, the modulation of alpha power was similar to the modulation of beta power only in the execution condition. There was practically no modulation in mental counting, whereas there was a sharp and relatively short-lived power decrease at the early stages of motor imagery. This was confirmed by a significant 2-way interaction between Condition and Time [*F*(2, 20) = 9.917, *p* < 0.001, η*_p_*^2^ = 0.002, see Figure 2B]. Post-hoc paired t-tests showed that there was a greater alpha power decrease in motor imagery compared to mental counting in Time 1 [*t*(20) = 3.53, *p* = 0.024], and a greater alpha power increase in motor imagery compared to mental counting in Time 3 [*t*(20) = 2.910, *p* = 0.036]. In addition, in Time 3, the alpha power increased in relation to the baseline in motor imagery, whereas there was still an alpha decrease in execution; this difference was statistically significant [*t*(20) = 3.414, *p* = 0.039].

A significant interaction between ROI and Time [*F*(2, 20) = 11.019, *p* < 0.001, η*_p_*^2^ = 0.016] revealed a stronger decrease of alpha power in the occipital ROI compared to the central [*t*(20) = 4.633, *p* < 0.001] and parietal [*t*(20) = 6.227, *p* < 0.001] ROIs in Time 2. In Time 4, a stronger increase of alpha power occurred over the occipital compared to the central [*t*(20) = 5.751, *p* < 0.001] and the parietal [*t*(20) = 4.893, *p* = 0.023] ROIs.

There was a significant interaction between Condition and ROI [*F*(2, 20) = 7.160, *p* < 0.001, η*_p_*^2^ = 0.583]. The amplitude of alpha power was larger in motor imagery compared to execution in the parietal [*t*(20) = 4.991, *p* < 0.001] and occipital [*t*(20) = 5.857, *p* < 0.001] ROIs.

A significant 3-way interaction [*F*(2, 20) = 2.881, *p* = 0.020, η*_p_*^2^ = 0.010] was further investigated in each ROI (see Figure 3, left panels). In the central ROI, there was a stronger decrease of alpha power in motor imagery compared to mental counting in Time 1 [*t*(20) = 3.693, *p* = 0.017]. In Time 3, the alpha power increased relative to baseline in motor imagery, but decreased in execution, showing a statistically significant difference [*t*(20) = 4.016, *p* = 0.005]. Similarly, in the parietal ROI, there was a stronger decrease of alpha power in motor imagery compared to mental counting in Time 1 [*t*(20) = 4.000, *p* = 0.012] and a stronger increase of alpha power in Time 4 [*t*(20) =2.818, *p* = 0.036]. In addition, in Time 3, the alpha power increased relative to baseline in motor imagery, and decreased for execution, although the difference was not statistically significant (*p* > 0.05). In the occipital ROI, there was a stronger increase of alpha power in Time 4 in motor imagery compared to mental counting [*t*(20) = 3.966, *p* = 0.007]. In addition, in Time 3, while the alpha power increased relative to baseline in motor imagery, and decreased in execution, there was no statistically significant difference (*p* > 0.05).

## 4 Discussion

The present study aimed to examine overlap and differences between the oscillatory brain activity related to motor imagery and actual execution of walking. The results showed a general match between the modulation of beta power regardless of whether participants were performing the actual execution of walking or the motor imagery of walking, whereas an equivalent correspondence did not emerge when participants were performing the control task of mental counting. Interestingly, the analysis also revealed clear evidence of differences between action execution and motor imagery. Specifically, a distinct pattern of beta activity was present during motor imagery, which was associated with a larger amplitude of beta power over central, parietal and occipital brain regions during motor imagery compared to actual walking. We also found that oscillatory activity in the alpha frequency band was characterized by a stronger power decrease at the beginning of the motor imagery task compared to during the actual execution and mental counting conditions. Therefore, the data suggests motor imagery and execution of naturalistic walking involve shared motor-cognitive activations, as shown by similar modulations in the alpha and beta frequency bands during motor imagery and action execution of walking, but that motor imagery requires additional cortical resources, indicated by the distinct pattern of more distributed brain activity involved during motor imagery compared to the other experimental conditions.

Consistent with the functional equivalence hypothesis (following motor simulation theory, *cf.* Jeannerod, 1994, 2001), our results demonstrated that motor imagery and actual execution of walking induced similar beta power decrease–increase dynamics, contrasting with the results observed during mental counting, suggesting that participants did not just count the steps mentally in the motor imagery condition. The decrease of beta oscillations during motor imagery signals the recruitment of neural circuits supporting the activation of motor-related information underlying the action representation of walking, which resembles typical cognitive mechanisms occurring during the preparation of overt motor responses (see Jeannerod, 2001; Glover and Baran, 2017). The role of decreased beta over the sensorimotor network has been defined as a mechanism that recruits neural processes to generate a motor output (Rhodes et al., 2018).

As such, we see neural overlap between the motor imagery of walking and actual walking in this motor-related beta decrease over the sensorimotor network. Beta power decrease has been previously reported during the kinaesthetic imagery of movement of body parts (i.e., hand, foot and tongue motor imagery) (Pfurtscheller and Neuper, 1997; Neuper et al., 2006; Pfurtscheller et al., 2006) and for motor imagery of skilled movements over sensorimotor areas (Pfurtscheller et al., 2002; Nakagawa et al., 2011; Di Nota et al., 2017). Furthermore, similar beta power decreases have been reported during motor imagery of simple and complex walking tasks (Putzolu et al., 2022), albeit with no comparison to execution of walking, and no inclusion of a control task, which lead to uncertainty about what was being measured. The neural overlap we see in the present study between the motor imagery of walking and actual walking evident in a motor-related beta decrease provides direct evidence for functional equivalence.

Further overlap in the beta oscillations in this study signaling functional equivalence is evident in a motor-related increase of power in the beta frequency band toward the end of the movement, both when imagining and executing walking. These power increases in the beta frequency band, also known as beta “rebound,” are typically observed over premotor and sensorimotor areas in the post movement phase (Pfurtscheller et al., 2005; Jurkiewicz et al., 2006; Pfurtscheller and Solis-Escalante, 2009). The beta rebound has been related to a resetting mechanism, reflecting processing that occurs at the end of a movement (Kilavik et al., 2013), and indicates the recruitment of neural circuits related to the reset (Engel and Fries, 2010), or similarly to inhibition (Salmelin et al., 1995), which is thought to be automatically recruited in motor imagery (Angelini et al., 2015). As a finding, it is remarkable that imagery of whole-body movement, that does not involve actual movement, generates this motor-related beta-rebound. That said, we also discuss differences related to the beta-rebound below.

The methodology and design of the current study, which allows for direct comparison between imagery and execution of whole-body movements, as well as having strong temporal resolution not only revealed overlap as presented above, but also motor-cognitive differences. As shown by our analysis, the beta power decrease seen in motor imagery task was shorter compared to actual execution of walking. However, as shown by the analysis, motor imagery required a larger recruitment of neural activation compared to the execution of walking, reflecting a greater effort when beginning to imagine the action without an actual overt motor output. Furthermore, the greater power decrease in the beta band observed in the initial phases of motor imagery compared to actual walking was evident over a broad area, including central, parietal and occipital cortical recording sites, suggesting the recruitment of brain regions included in the sensorimotor network (Solodkin et al., 2004).

Recent meta-analyses have revealed that motor imagery involves a greater range of brain areas which are not activated during actual execution (Hétu et al., 2013; Hardwick et al., 2018). This broader neural network, that includes motor brain areas, encompasses parietal and occipital regions, which are known to play a key role in motor planning and in the processing of visuo-spatial information during spatial navigation (Andersen, 1997; Ino et al., 2002; Cui and Andersen, 2007; Julian et al., 2016). According to recent accounts, a function of motor imagery is to promote action predictions supporting the processing of motor and sensory information in order to guide behavior (Bubic et al., 2010; Ridderinkhof and Brass, 2015; O’Shea and Moran, 2017). Consistent with these findings, our results showed a larger parietal and occipital activation when participants imagined themselves walking, compared to when they actually walked and when they simply mentally counted. These results indicate that the imagery condition required the active maintenance of action simulation as well as a dynamic integration of visual and kinaesthetic sensory information associated with the motor imagery of one’s body moving in the environment (Krüger et al., 2020, 2022). Taken together, therefore, current findings suggest that motor imagery involves cognitive mechanisms that go beyond the mere encoding of motor information and point toward the multisensory nature of motor imagery (Dahm, 2020; Kraeutner et al., 2020; Krüger et al., 2022; Frank et al., 2023).

Differences in the temporal dynamics were also evident at the end of the movement. The results showed an increase of power in the beta frequency band toward the end of the walking movement in all three tasks, but with an overall stronger effect in the motor imagery condition. The beta power increase was apparent at different times across the three experimental conditions, with two peaks occurring during the last two time intervals in the motor imagery condition. From a temporal perspective, it was notable that the first of these peaks coincided with the beta peak in the mental counting condition, whereas the second peak coincided with the beta peak in the actual execution condition. Therefore, the beta-rebound appears to occur twice in the motor imagery condition, but for different reasons. The first “early” increase of beta power might indicate the end of the mental counting of the steps as part of imagery, whereas the second “delayed” increase of beta power appears to be related to the reset of the motor representation, occurring at the same time as the end of walking execution. Notably, it is the neural overlap in the motor-related beta-rebound between motor imagery and actual execution of whole-body walking movements here that is most remarkable.

These data also indicated a different alpha power modulation between the three experimental conditions. It is well known that during movement preparation and execution, a power decrease in the alpha and beta frequency bands occurs over sensorimotor areas (Pfurtscheller and Berghold, 1989; Leocani et al., 2001; Kaiser et al., 2003). In the present study, we found a sustained alpha decrease during actual execution, consistent with previous findings of walking (Gwin et al., 2011; Bulea et al., 2015; Bradford et al., 2016). Similar to our findings in the beta band, the results showed a prominent alpha power decrease in the motor imagery condition, but only at the beginning of the imagined walking, whereas there was no evidence of a modulation during the mental counting condition. Previous literature has suggested that such decreases in alpha power reflect the allocation of attention toward relevant task-related information, such as visual information (Foxe and Snyder, 2011; Brinkman et al., 2014) and furthermore, providing an index of cortical activation, in contrast to alpha increase of power which reflects cortical inhibition (the “idling” state, Pfurtscheller et al., 1996). From this perspective, the strong alpha decrease seen at the beginning of motor imagery could indicate either the initial allocation of attention toward relevant features of the action being simulated or to the active processing of motor and sensory information (Foxe and Snyder, 2011; Brinkman et al., 2014). Alpha suppression has been observed during motor imagery and movement observation (Pfurtscheller and Da Silva, 1999; Jurkiewicz et al., 2006; de Lange et al., 2008), and also specifically during motor imagery of simple and complex gait (Putzolu et al., 2022). The temporal specificity of the neural activity during motor imagery, and the contrast with actual execution of whole-body movements in this study, signals that motor imagery requires the allocation of attention, enabling active processing of motor and sensory information in order to simulate action.

Overall, the present results indicate that motor imagery and actual execution of whole-body walking movement share common neural features, as predicted by the functional equivalence hypothesis. Shared features occur during the early stages of walking action execution and motor imagery, likely reflecting the initiation of an action plan (Jeannerod, 1994, 2001; Decety, 1996). Overlap was also evident in the beta-rebound at the end of the action, suggesting motor imagery and execution of whole-body movements involve similar resetting or inhibition of motor processes (Engel and Fries, 2010). These similar mechanisms are reflected in beta and alpha power modulation during motor imagery, which resemble action execution patterns. The motor-cognitive processes of whole-body motor imagery was furthermore revealed through the differences between motor imagery and the actual execution of walking in this study. The data indicate that motor imagery elicits a wider range of brain activity, especially at the beginning of the task, compared to execution. The broader pattern of neural activation indicates that the cognitive mechanisms of motor imagery go beyond the mere encoding of motor-related information, and that motor imagery places greater cognitive demands than execution presumably due to the requirement to initiate and sustain a deliberate mental simulation of action. This is relevant information, for example when considering that stroke patients do not necessarily benefit from motor imagery as part of their neuro-rehabilitation (Ietswaart et al., 2011).

This is the first report of brain activity recorded during naturalistic full-body movement contrasted with the imagery of walking. Using the same imaging methods in both conditions, direct comparison between the neural correlates of execution and motor imagery of walking could be made. Furthermore, an upright body position was maintained during imagery to match the posture of actual walking. These design features were not present in the one other neuroimaging study (La Fougere et al., 2010) comparing imagery and execution of walking. EEG recordings furthermore allowed for the characterization of the temporal aspects of brain dynamics, to disentangle the cognitive processes occurring during motor imagery and action execution at different time points. As such, this is the first study that showed directly that motor imagery and execution of naturalistic walking involves shared motor-cognitive activations, and that motor imagery requires additional cortical resources.

## Data availability statement

The data supporting the findings of this study are available at https://osf.io/rth4c/?view_only=bea46044373d43dd95fde4bb6fa78b08.

## Ethics statement

The studies involving humans were approved by the Ethics Committee of University of Stirling. The studies were conducted in accordance with the local legislation and institutional requirements. The participants provided their written informed consent to participate in this study.

## Author contributions

MM: Conceptualization, Data curation, Formal analysis, Investigation, Methodology, Project administration, Validation, Writing – original draft, Writing – review & editing. DK: Conceptualization, Data curation, Formal analysis, Methodology, Supervision, Validation, Writing – original draft, Writing – review & editing. ME: Conceptualization, Data curation, Methodology, Supervision, Validation, Writing – original draft, Writing – review & editing. DD: Conceptualization, Data curation, Methodology, Supervision, Validation, Writing – original draft, Writing – review & editing. MI: Conceptualization, Data curation, Funding acquisition, Investigation, Methodology, Resources, Supervision, Validation, Writing – original draft, Writing – review & editing.

## References

[ref1] Andersen R. A. (1997). Multimodal integration for the representation of space in the posterior parietal cortex. Philos. Trans. R. Soc. Lond. B Biol. Sci. 352, 1421–1428. doi: 10.1098/rstb.1997.0128, PMID: 9368930 PMC1692052

[ref9001] Angelini M. Calbi M. Ferrari A. Sbriscia-Fioretti B. Franca M. Gallese V.. (2015). Motor inhibition during overt and covert actions: an electrical neuroimaging study. PloS one, 10, e0126800. 26000451 10.1371/journal.pone.0126800 PMC4441499

[ref2] Bakker M. De Lange F. P. Stevens J. A. Toni I. Bloem B. R. (2007). Motor imagery of gait: a quantitative approach. Exp. Brain Res. 179, 497–504. doi: 10.1007/s00221-006-0807-x 17211663

[ref3] Barton J. Pretty J. (2010). What is the best dose of nature and green exercise for improving mental health? A multi-study analysis. Environ. Sci. Technol. 44, 3947–3955. doi: 10.1021/es903183r 20337470

[ref4] Bradford J. C. Lukos J. R. Ferris D. P. (2016). Electrocortical activity distinguishes between uphill and level walking in humans. J. Neurophysiol. 115, 958–966. doi: 10.1152/jn.00089.2015, PMID: 26683062

[ref5] Brinkman L. Stolk A. Dijkerman H. C. de Lange F. P. Toni I. (2014). Distinct roles for alpha-and beta-band oscillations during mental simulation of goal-directed actions. J. Neurosci. 34, 14783–14792. doi: 10.1523/JNEUROSCI.2039-14.2014, PMID: 25355230 PMC4212072

[ref6] Bubic A. Von Cramon D. Y. Schubotz R. I. (2010). Prediction, cognition and the brain. Front. Hum. Neurosci. 4:1094. doi: 10.3389/fnhum.2010.00025 PMC2904053 20631856

[ref7] Bulea T. C. Kim J. Damiano D. L. Stanley C. J. Park H. S. (2015). Prefrontal, posterior parietal and sensorimotor network activity underlying speed control during walking. Front. Hum. Neurosci. 9:247. doi: 10.3389/fnhum.2015.00247 26029077 PMC4429238

[ref8] Cassim F. Szurhaj W. Sediri H. Devos D. Bourriez J. L. Poirot I.. (2000). Brief and sustained movements: differences in event-related (de) synchronization (ERD/ERS) patterns. Clin. Neurophysiol. 111, 2032–2039. doi: 10.1016/S1388-2457(00)00455-7 11068239

[ref9] Choi K. Cichocki A. (2008). “Control of a wheelchair by motor imagery in real time” in Intelligent data engineering and automated learning–IDEAL 2008: 9th international conference Daejeon, South Korea, November 2–5, 2008 proceedings 9 (Berlin Heidelberg: Springer), 330–337.

[ref10] Cochrane Stroke Group Barclay R. E. Stevenson T. J. Poluha W. Semenko B. Schubert J. (2020). Mental practice for treating upper extremity deficits in individuals with hemiparesis after stroke. Cochrane Database Syst. Rev. 2020:CD005950. doi: 10.1002/14651858.CD005950.pub5, PMID: 32449959 PMC7387111

[ref11] Conson M. Mazzarella E. Trojano L. (2011). Self-touch affects motor imagery: a study on posture interference effect. Exp. Brain Res. 215, 115–122. doi: 10.1007/s00221-011-2877-7 21947174

[ref12] Cui H. Andersen R. A. (2007). Posterior parietal cortex encodes autonomously selected motor plans. Neuron 56, 552–559. doi: 10.1016/j.neuron.2007.09.031 17988637 PMC2651089

[ref13] Daeglau M. Wallhoff F. Debener S. Condro I. S. Kranczioch C. Zich C. (2020). Challenge accepted? Individual performance gains for motor imagery practice with humanoid robotic EEG neurofeedback. Sensors 20:1620. doi: 10.3390/s20061620, PMID: 32183285 PMC7146190

[ref14] Dahm S. F. (2020). On the assessment of motor imagery ability: a research commentary. Imagin. Cogn. Pers. 39, 397–408. doi: 10.1177/0276236619836091

[ref15] de Lange F. P. Helmich R. C. Toni I. (2006). Posture influences motor imagery: an fMRI study. Neuroimage 33:609. doi: 10.1016/j.neuroimage.2006.07.017 16959501

[ref16] de Lange F. P. Jensen O. Bauer M. Toni I. (2008). Interactions between posterior gamma and frontal alpha/beta oscillations during imagined actions. Front. Hum. Neurosci. 2:269. doi: 10.3389/neuro.09.007.2008 PMC2572199 18958208

[ref17] Decety J. (1996). The neurophysiological basis of motor imagery. Behav. Brain Res. 77, 45–52. doi: 10.1016/0166-4328(95)00225-1, PMID: 8762158

[ref18] Decety J. Jeannerod M. (1995). Mentally simulated movements in virtual reality: does Fitt's law hold in motor imagery? Behav. Brain Res. 72, 127–134. doi: 10.1016/0166-4328(96)00141-6 8788865

[ref20] Decety J. Michel F. (1989). Comparative analysis of actual and mental movement times in two graphic tasks. Brain Cogn. 11, 87–97. doi: 10.1016/0278-2626(89)90007-9, PMID: 2789819

[ref21] Delorme A. Makeig S. (2004). EEGLAB: an open source toolbox for analysis of single-trial EEG dynamics including independent component analysis. J. Neurosci. Methods 134, 9–21. doi: 10.1016/j.jneumeth.2003.10.009, PMID: 15102499

[ref22] Di Nota P. M. Chartrand J. M. Levkov G. R. Montefusco-Siegmund R. DeSouza J. F. (2017). Experience-dependent modulation of alpha and beta during action observation and motor imagery. BMC Neurosci. 18, 1–14. doi: 10.1186/s12868-017-0349-0 28264664 PMC5340035

[ref23] Dijkerman H. C. Ietswaart M. Johnston M. (2010). “Motor imagery and the rehabilitation of movement disorders: an overview” in The neurophysiological foundations of mental and motor imagery. eds. Guillot A. Collet C., Oxford University Press. 127–144.4.

[ref9002] Engel A. K. Fries P. (2010). Beta-band oscillations—signalling the status quo?. Current opinion in neurobiology, 20, 156–165. 20359884 10.1016/j.conb.2010.02.015

[ref24] Foxe J. J. Snyder A. C. (2011). The role of alpha-band brain oscillations as a sensory suppression mechanism during selective attention. Front. Psychol. 2:154. doi: 10.3389/fpsyg.2011.00154 21779269 PMC3132683

[ref25] Frank C. Kraeutner S. N. Rieger M. Boe S. G. (2023). Learning motor actions via imagery—perceptual or motor learning? Psychol. Res., 1–13. doi: 10.1007/s00426-022-01787-4 PMC11315805 36680584

[ref26] Glover S. Baran M. (2017). The motor-cognitive model of motor imagery: evidence from timing errors in simulated reaching and grasping. J. Exp. Psychol. Hum. Percept. Perform. 43, 1359–1375. doi: 10.1037/xhp0000389 28368162

[ref27] Grezes J. Decety J. (2001). Functional anatomy of execution, mental simulation, observation, and verb generation of actions: a meta-analysis. Hum. Brain Mapp. 12, 1–19. doi: 10.1002/1097-0193(200101)12:1<1::AID-HBM10>3.0.CO;2-V, PMID: 11198101 PMC6872039

[ref28] Guillot A. Collet C. (2008). Construction of the motor imagery integrative model in sport: a review and theoretical investigation of motor imagery use. Int. Rev. Sport Exerc. Psychol. 1, 31–44. doi: 10.1080/17509840701823139

[ref29] Gwin J. T. Gramann K. Makeig S. Ferris D. P. (2011). Electrocortical activity is coupled to gait cycle phase during treadmill walking. Neuroimage 54, 1289–1296. doi: 10.1016/j.neuroimage.2010.08.066, PMID: 20832484

[ref9004] Hamacher D. Herold F. Wiegel P. Hamacher D. Schega L. (2015). Brain activity during walking: a systematic review. Neuroscience & Biobehavioral Reviews,, 57, 310–327. 26306029 10.1016/j.neubiorev.2015.08.002

[ref30] Hardwick R. M. Caspers S. Eickhoff S. B. Swinnen S. P. (2018). Neural correlates of action: comparing meta-analyses of imagery, observation, and execution. Neurosci. Biobehav. Rev. 94, 31–44. doi: 10.1016/j.neubiorev.2018.08.003, PMID: 30098990

[ref31] Hashimoto Y. Ushiba J. (2013). EEG-based classification of imaginary left and right foot movements using beta rebound. Clin. Neurophysiol. 124, 2153–2160. doi: 10.1016/j.clinph.2013.05.006 23757379

[ref32] Hétu S. Grégoire M. Saimpont A. Coll M. P. Eugène F. Michon P. E.. (2013). The neural network of motor imagery: an ALE meta-analysis. Neurosci. Biobehav. Rev. 37, 930–949. doi: 10.1016/j.neubiorev.2013.03.017, PMID: 23583615

[ref33] Ietswaart M. Johnston M. Dijkerman H. C. Joice S. Scott C. L. MacWalter R. S.. (2011). Mental practice with motor imagery in stroke recovery: randomized controlled trial of efficacy. Brain 134, 1373–1386. doi: 10.1093/brain/awr077, PMID: 21515905 PMC3097892

[ref34] Ino T. Inoue Y. Kage M. Hirose S. Kimura T. Fukuyama H. (2002). Mental navigation in humans is processed in the anterior bank of the parieto-occipital sulcus. Neurosci. Lett. 322, 182–186. doi: 10.1016/S0304-3940(02)00019-8 11897168

[ref9006] Jahn K. Deutschländer A. Stephan T. Strupp M. Wiesmann M. Brandt T. (2004). Brain activation patterns during imagined stance and locomotion in functional magnetic resonance imaging. Neuroimage, 22, 1722–1731. 15275928 10.1016/j.neuroimage.2004.05.017

[ref9007] Jahn K. Deutschländer A. Stephan T. Kalla R. Wiesmann M. Strupp M.. (2008). Imaging human supraspinal locomotor centers in brainstem and cerebellum. Neuroimage, 39, 786–792. 18029199 10.1016/j.neuroimage.2007.09.047

[ref35] Jeannerod M. (1994). The representing brain: neural correlates of motor intention and imagery. Behav. Brain Sci. 17, 187–202. doi: 10.1017/S0140525X00034026

[ref36] Jeannerod M. (2001). Neural simulation of action: a unifying mechanism for motor cognition. NeuroImage 14, S103–S109. doi: 10.1006/nimg.2001.0832 11373140

[ref37] Julian J. B. Ryan J. Hamilton R. H. Epstein R. A. (2016). The occipital place area is causally involved in representing environmental boundaries during navigation. Curr. Biol. 26, 1104–1109. doi: 10.1016/j.cub.2016.02.066, PMID: 27020742 PMC5565511

[ref38] Jurkiewicz M. T. Gaetz W. C. Bostan A. C. Cheyne D. (2006). Post-movement beta rebound is generated in motor cortex: evidence from neuromagnetic recordings. NeuroImage 32, 1281–1289. doi: 10.1016/j.neuroimage.2006.06.005 16863693

[ref39] Kaiser J. Ulrich R. Lutzenberger W. (2003). Dynamics of sensorimotor cortex activation to spatial sounds precueing ipsi-versus contralateral manual responses. Cogn. Brain Res. 17, 573–583. doi: 10.1016/S0926-6410(03)00171-X, PMID: 14561446

[ref40] Kilavik B. E. Zaepffel M. Brovelli A. MacKay W. A. Riehle A. (2013). The ups and downs of beta oscillations in sensorimotor cortex. Exp. Neurol. 245, 15–26. doi: 10.1016/j.expneurol.2012.09.014, PMID: 23022918

[ref41] Klimesch W. (2012). Alpha-band oscillations, attention, and controlled access to stored information. Trends Cogn. Sci. 16, 606–617. doi: 10.1016/j.tics.2012.10.007, PMID: 23141428 PMC3507158

[ref42] Kline A. Ghiroaga C. G. Pittman D. Goodyear B. Ronsky J. (2021). EEG differentiates left and right imagined lower limb movement. Gait Posture 84, 148–154. doi: 10.1016/j.gaitpost.2020.11.014, PMID: 33340844

[ref43] Kraeutner S. N. Eppler S. N. Stratas A. Boe S. G. (2020). Generate, maintain, manipulate? Exploring the multidimensional nature of motor imagery. Psychol. Sport Exerc. 48:101673. doi: 10.1016/j.psychsport.2020.101673

[ref44] Kranczioch C. Zich C. Schierholz I. Sterr A. (2014). Mobile EEG and its potential to promote the theory and application of imagery-based motor rehabilitation. Int. J. Psychophysiol. 91, 10–15. doi: 10.1016/j.ijpsycho.2013.10.004 24144637

[ref45] Krüger B. Hegele M. Rieger M. (2022). The multisensory nature of human action imagery. Psychol. Res., 1–13. doi: 10.1007/s00426-022-01771-y PMC11315721 36441293

[ref46] Krüger B. Zabicki A. Grosse L. Naumann T. Munzert J. (2020). Sensory features of mental images in the framework of human actions. Conscious. Cogn. 83:102970. doi: 10.1016/j.concog.2020.102970, PMID: 32540626

[ref9010] Ladouce S. Donaldson D. I. Dudchenko P. A. Ietswaart M. (2017). Understanding minds in real-world environments: toward a mobile cognition approach. Frontiers in human neuroscience, 10, 694. 28127283 10.3389/fnhum.2016.00694 PMC5226959

[ref47] La Fougere C. Zwergal A. Rominger A. Förster S. Fesl G. Dieterich M.. (2010). Real versus imagined locomotion: a [18F]-FDG PET-fMRI comparison. Neuroimage 50, 1589–1598. doi: 10.1016/j.neuroimage.2009.12.060, PMID: 20034578

[ref48] LaFleur K. Cassady K. Doud A. Shades K. Rogin E. He B. (2013). Quadcopter control in three-dimensional space using a noninvasive motor imagery-based brain–computer interface. J. Neural Eng. 10:046003. doi: 10.1088/1741-2560/10/4/046003 23735712 PMC3839680

[ref49] Leeb R. Friedman D. Müller-Putz G. R. Scherer R. Slater M. Pfurtscheller G. (2007). Self-paced (asynchronous) BCI control of a wheelchair in virtual environments: a case study with a tetraplegic. Comput. Intell. Neurosci. 2007, 1–8. doi: 10.1155/2007/79642, PMID: 18368142 PMC2272302

[ref50] Leocani L. Toro C. Zhuang P. Gerloff C. Hallett M. (2001). Event-related desynchronization in reaction time paradigms: a comparison with event-related potentials and corticospinal excitability. Clin. Neurophysiol. 112, 923–930. doi: 10.1016/S1388-2457(01)00530-2, PMID: 11336910

[ref51] Lorey B. Bischoff M. Pilgramm S. Stark R. Munzert J. Zentgraf K. (2009). The embodied nature of motor imagery: the influence of posture and perspective. Exp. Brain Res. 194, 233–243. doi: 10.1007/s00221-008-1693-1, PMID: 19139856

[ref52] Macuga K. L. Frey S. H. (2012). Neural representations involved in observed, imagined, and imitated actions are dissociable and hierarchically organized. NeuroImage 59, 2798–2807. doi: 10.1016/j.neuroimage.2011.09.083, PMID: 22005592 PMC3254825

[ref53] Macuga K. L. Papailiou A. P. Frey S. H. (2012). Motor imagery of tool use: relationship to actual use and adherence to Fitts’ law across tasks. Exp. Brain Res. 218, 169–179. doi: 10.1007/s00221-012-3004-0, PMID: 22294026 PMC3351569

[ref9003] Makeig S. Bell A. Jung T. P. Sejnowski T. J. (1995). Independent component analysis of electroencephalographic data. Advances in neural information processing systems, 8.

[ref54] Malouin F. Jackson P. L. Richards C. L. (2013). Towards the integration of mental practice in rehabilitation programs. A critical review. Front. Hum. Neurosci. 7:576. doi: 10.3389/fnhum.2013.00576 24065903 PMC3776942

[ref55] Malouin F. Richards C. L. (2010). Mental practice for relearning locomotor skills. Phys. Ther. 90, 240–251. doi: 10.2522/ptj.20090029 20022993

[ref56] Menicucci D. Di Gruttola F. Cesari V. Gemignani A. Manzoni D. Sebastiani L. (2020). Task-independent electrophysiological correlates of motor imagery ability from kinaesthetic and visual perspectives. Neuroscience 443, 176–187. doi: 10.1016/j.neuroscience.2020.07.038 32736068

[ref57] Mizuguchi N. Nakamura M. Kanosue K. (2017). Task-dependent engagements of the primary visual cortex during kinesthetic and visual motor imagery. Neurosci. Lett. 636, 108–112. doi: 10.1016/j.neulet.2016.10.064, PMID: 27826015

[ref59] Mulder T. (2007). Motor imagery and action observation: cognitive tools for rehabilitation. J. Neural Transm. 114, 1265–1278. doi: 10.1007/s00702-007-0763-z, PMID: 17579805 PMC2797860

[ref9005] Müller-Putz G. R. Kaiser V. Solis-Escalante T. Pfurtscheller G. (2010). Fast set-up asynchronous brain-switch based on detection of foot motor imagery in 1-channel EEG. Medical & biological engineering & computing, 48, 229–233. 20052556 10.1007/s11517-009-0572-7

[ref60] Mustile M. Kourtis D. Edwards M. G. Donaldson D. I. Ietswaart M. (2022). The neural response is heightened when watching a person approaching compared to walking away: evidence for dynamic social neuroscience. Neuropsychologia 175:108352. doi: 10.1016/j.neuropsychologia.2022.108352, PMID: 36007672

[ref61] Nakagawa K. Aokage Y. Fukuri T. Kawahara Y. Hashizume A. Kurisu K.. (2011). Neuromagnetic beta oscillation changes during motor imagery and motor execution of skilled movements. Neuroreport 22, 217–222. doi: 10.1097/WNR.0b013e328344b480, PMID: 21386697

[ref62] Neuper C. Pfurtscheller G. (2001). Event-related dynamics of cortical rhythms: frequency-specific features and functional correlates. Int. J. Psychophysiol. 43, 41–58. doi: 10.1016/S0167-8760(01)00178-7, PMID: 11742684

[ref9008] Neuper C. Wörtz M. Pfurtscheller G. (2006). ERD/ERS patterns reflecting sensorimotor activation and deactivation. Progress in brain research, 159, 211–222 17071233 10.1016/S0079-6123(06)59014-4

[ref9009] Neuper C. Pfurtscheller G. Guillot A. Collet C. (2010). Electroencephalographic characteristics during motor imagery. The neurophysiological foundations of mental and motor imagery, 65–81.

[ref63] O’Shea H. Moran A. (2017). Does motor simulation theory explain the cognitive mechanisms underlying motor imagery? A critical review. Front. Hum. Neurosci. 11:72. doi: 10.3389/fnhum.2017.00072 28261079 PMC5313484

[ref64] Parsons L. M. (1994). Temporal and kinematic properties of motor behavior reflected in mentally simulated action. J. Exp. Psychol. Hum. Percept. Perform. 20, 709–730. doi: 10.1037//0096-1523.20.4.709, PMID: 8083630

[ref65] Pfurtscheller G. (1999). “Event-related desynchronization and related oscillatory phenomena of the brain” in Handbook of electroencephalography and clinical neurophysiology. Elsevier.

[ref66] Pfurtscheller G. Berghold A. (1989). Patterns of cortical activation during planning of voluntary movement. Electroencephalogr. Clin. Neurophysiol. 72, 250–258. doi: 10.1016/0013-4694(89)90250-2 2465128

[ref67] Pfurtscheller G. Da Silva F. L. (1999). Event-related EEG/MEG synchronization and desynchronization: basic principles. Clin. Neurophysiol. 110, 1842–1857. doi: 10.1016/S1388-2457(99)00141-8, PMID: 10576479

[ref68] Pfurtscheller G. Leeb R. Keinrath C. Friedman D. Neuper C. Guger C.. (2006). Walking from thought. Brain Res. 1071, 145–152. doi: 10.1016/j.brainres.2005.11.083 16405926

[ref69] Pfurtscheller G. Müller G. R. Pfurtscheller J. Gerner H. J. Rupp R. (2003). ‘Thought’–control of functional electrical stimulation to restore hand grasp in a patient with tetraplegia. Neurosci. Lett. 351, 33–36. doi: 10.1016/S0304-3940(03)00947-9, PMID: 14550907

[ref70] Pfurtscheller G. Neuper C. (1997). Motor imagery activates primary sensorimotor area in humans. Neurosci. Lett. 239, 65–68. doi: 10.1016/S0304-3940(97)00889-6 9469657

[ref71] Pfurtscheller G. Neuper C. Brunner C. Da Silva F. L. (2005). Beta rebound after different types of motor imagery in man. Neurosci. Lett. 378, 156–159. doi: 10.1016/j.neulet.2004.12.034, PMID: 15781150

[ref72] Pfurtscheller G. Neuper C. Flotzinger D. Pregenzer M. (1997). EEG-based discrimination between imagination of right and left hand movement. Electroencephalogr. Clin. Neurophysiol. 103, 642–651. doi: 10.1016/S0013-4694(97)00080-1 9546492

[ref73] Pfurtscheller G. Solis-Escalante T. (2009). Could the beta rebound in the EEG be suitable to realize a “brain switch”? Clin. Neurophysiol. 120, 24–29. doi: 10.1016/j.clinph.2008.09.027, PMID: 19028138

[ref74] Pfurtscheller G. Stancak A. Jr. Neuper C. (1996). Event-related synchronization (ERS) in the alpha band—an electrophysiological correlate of cortical idling: a review. Int. J. Psychophysiol. 24, 39–46. doi: 10.1016/S0167-8760(96)00066-9 8978434

[ref75] Pfurtscheller G. Woertz M. Müller G. Wriessnegger S. Pfurtscheller K. (2002). Contrasting behavior of beta event-related synchronization and somatosensory evoked potential after median nerve stimulation during finger manipulation in man. Neurosci. Lett. 323, 113–116. doi: 10.1016/S0304-3940(02)00119-2, PMID: 11950506

[ref76] Pion-Tonachini L. Kreutz-Delgado K. Makeig S. (2019). ICLabel: an automated electroencephalographic independent component classifier, dataset, and website. NeuroImage 198, 181–197. doi: 10.1016/j.neuroimage.2019.05.026, PMID: 31103785 PMC6592775

[ref77] Porro C. A. Francescato M. P. Cettolo V. Diamond M. E. Baraldi P. Zuiani C.. (1996). Primary motor and sensory cortex activation during motor performance and motor imagery: a functional magnetic resonance imaging study. J. Neurosci. 16, 7688–7698. doi: 10.1523/JNEUROSCI.16-23-07688.1996, PMID: 8922425 PMC6579073

[ref78] Putzolu M. Samogin J. Cosentino C. Mezzarobba S. Bonassi G. Lagravinese G.. (2022). Neural oscillations during motor imagery of complex gait: an HdEEG study. Sci. Rep. 12:4314. doi: 10.1038/s41598-022-07511-x, PMID: 35279682 PMC8918338

[ref79] Rhodes E. Gaetz W. C. Marsden J. Hall S. D. (2018). Transient alpha and beta synchrony underlies preparatory recruitment of directional motor networks. J. Cogn. Neurosci. 30, 867–875. doi: 10.1162/jocn_a_01250, PMID: 29488848

[ref80] Ridderinkhof K. R. Brass M. (2015). How kinesthetic motor imagery works: a predictive-processing theory of visualization in sports and motor expertise. J. Physiol. 109, 53–63. doi: 10.1016/j.jphysparis.2015.02.003, PMID: 25817985

[ref81] Ruffino C. Papaxanthis C. Lebon F. (2017). Neural plasticity during motor learning with motor imagery practice: review and perspectives. Neuroscience 341, 61–78. doi: 10.1016/j.neuroscience.2016.11.023 27890831

[ref82] Sackett R. S. (1934). The influence of symbolic rehearsal upon the retention of a maze habit. J. Gen. Psychol. 10, 376–398. doi: 10.1080/00221309.1934.9917742

[ref9011] Sackett R. S. (1935). The relationship between amount of symbolic rehearsal and retention of a maze habit. The Journal of General Psychology, 13, 113–130.

[ref83] Saimpont A. Malouin F. Tousignant B. Jackson P. L. (2012). The influence of body configuration on motor imagery of walking in younger and older adults. Neuroscience 222, 49–57. doi: 10.1016/j.neuroscience.2012.06.066, PMID: 22796073

[ref84] Salenius S. Kajola M. Thompson W. L. Kosslyn S. Hari R. (1995). Reactivity of magnetic parieto-occipital alpha rhythm during visual imagery. Electroencephalogr. Clin. Neurophysiol. 95, 453–462. doi: 10.1016/0013-4694(95)00155-7 8536574

[ref85] Salmelin R. Hámáaláinen M. Kajola M. Hari R. (1995). Functional segregation of movement-related rhythmic activity in the human brain. NeuroImage 2, 237–243. doi: 10.1006/nimg.1995.1031, PMID: 9343608

[ref9012] Sirigu A. Cohen L. Duhamel J. R. Pillon B. Dubois B. Agid Y.. (1995). Congruent unilateral impairments for real and imagined hand movements. Neuroreport, 6, 997–1001. 7632907 10.1097/00001756-199505090-00012

[ref86] Sharma N. Baron J. C. (2013). Does motor imagery share neural networks with executed movement: a multivariate fMRI analysis. Front. Hum. Neurosci. 7:564. doi: 10.3389/fnhum.2013.00564 24062666 PMC3771114

[ref9013] Solis-Escalante T. Müller-Putz G. Pfurtscheller G. (2008). Overt foot movement detection in one single Laplacian EEG derivation. Journal of neuroscience methods, 175, 148–153. 18761037 10.1016/j.jneumeth.2008.07.019

[ref9014] Solis-Escalante T. Müller-Putz G. R. Pfurtscheller G. Neuper C. (2012). Cue-induced beta rebound during withholding of overt and covert foot movement. Clinical Neurophysiology, 123, 1182–1190. 22349305 10.1016/j.clinph.2012.01.013

[ref9015] Solodkin A. Hlustik P. Chen E. E. Small S. L. (2004). Fine modulation in network activation during motor execution and motor imagery. Cerebral cortex, 14, 1246–1255. 15166100 10.1093/cercor/bhh086

[ref9016] Stančák Jr A. Riml A. Pfurtscheller G. (1997). The effects of external load on movement-related changes of the sensorimotor EEG rhythms. Electroencephalography and Clinical Neurophysiology, 102, 495–504. 9216482 10.1016/s0013-4694(96)96623-0

[ref87] Tzagarakis C. Ince N. F. Leuthold A. C. Pellizzer G. (2010). Beta-band activity during motor planning reflects response uncertainty. J. Neurosci. 30, 11270–11277. doi: 10.1523/JNEUROSCI.6026-09.2010 20739547 PMC6633326

[ref88] Williams S. E. Cumming J. Ntoumanis N. Nordin-Bates S. M. Ramsey R. Hall C. (2012). Further validation and development of the movement imagery questionnaire. J. Sport Exerc. Psychol. 34, 621–646. doi: 10.1123/jsep.34.5.621, PMID: 23027231

[ref89] Wolpaw J. R. Birbaumer N. McFarland D. J. Pfurtscheller G. Vaughan T. M. (2002). Brain–computer interfaces for communication and control. Clin. Neurophysiol. 113, 767–791. doi: 10.1016/S1388-2457(02)00057-3 12048038

[ref90] Xie S. Kaiser D. Cichy R. M. (2020). Visual imagery and perception share neural representations in the alpha frequency band. Curr. Biol. 30, 2621–2627.e5. doi: 10.1016/j.cub.2020.04.074, PMID: 32531274 PMC7342016

